# Supplementary material for: Resolving the full spectrum of human genome variation using Linked-Reads
Source: Genome Res. 2019 Apr;29(4):635–45. doi: 10.1101/gr.234443.118 (PMC6442396; doi:10.1101/gr.234443.118)
Supplement: Supplemental Material [file supp_gr.234443.118_Supplemental_longranger-2.2.zip › longranger-2.2.tar.bz2!/lr2.2-public/lib/stan/cmdstan/stan/doc/api/html/structstan_1_1gm_1_1program__grammar.html]

Stan: stan::gm::program\_grammar< Iterator > Struct Template Reference


|  |  |
| --- | --- |
|  | Stan  2.5.0  probability, sampling & optimization |


- Main Page
- Related Pages
- Namespaces
- Classes
- Files

- Class List
- Class Index
- Class Hierarchy
- Class Members

All Classes Namespaces Files Functions Variables Typedefs Enumerations Enumerator Friends Macros Pages

- stan
- gm
- program\_grammar

Public Member Functions |
Public Attributes |
List of all members

stan::gm::program\_grammar< Iterator > Struct Template Reference

`#include <program_grammar.hpp>`

Inheritance diagram for stan::gm::program\_grammar< Iterator >:

|  |  |
| --- | --- |
| Public Member Functions | |
|  | program\_grammar (const std::string &model\_name) |
|  | |

|  |  |
| --- | --- |
| Public Attributes | |
| std::string | model\_name\_ |
|  | |
| variable\_map | var\_map\_ |
|  | |
| std::stringstream | error\_msgs\_ |
|  | |
| expression\_grammar< Iterator > | expression\_g |
|  | |
| var\_decls\_grammar< Iterator > | var\_decls\_g |
|  | |
| statement\_grammar< Iterator > | statement\_g |
|  | |
| functions\_grammar< Iterator > | functions\_g |
|  | |
| boost::spirit::qi::rule  < Iterator, std::vector  < var\_decl >  ), whitespace\_grammar  < Iterator > > | data\_var\_decls\_r |
|  | |
| boost::spirit::qi::rule  < Iterator, std::pair  < std::vector< var\_decl >  , std::vector< statement >  >), whitespace\_grammar  < Iterator > > | derived\_data\_var\_decls\_r |
|  | |
| boost::spirit::qi::rule  < Iterator, std::pair  < std::vector< var\_decl >  , std::vector< statement >  >), whitespace\_grammar  < Iterator > > | derived\_var\_decls\_r |
|  | |
| boost::spirit::qi::rule  < Iterator, std::pair  < std::vector< var\_decl >  , std::vector< statement >  >), whitespace\_grammar  < Iterator > > | generated\_var\_decls\_r |
|  | |
| boost::spirit::qi::rule  < Iterator, statement(),   whitespace\_grammar< Iterator > > | model\_r |
|  | |
| boost::spirit::qi::rule  < Iterator, std::vector  < var\_decl >  ), whitespace\_grammar  < Iterator > > | param\_var\_decls\_r |
|  | |
| boost::spirit::qi::rule  < Iterator, program(),   whitespace\_grammar< Iterator > > | program\_r |
|  | |

## Detailed Description

### template<typename Iterator> struct stan::gm::program\_grammar< Iterator >

Definition at line 22 of file program\_grammar.hpp.

## Constructor & Destructor Documentation

template<typename Iterator >

|  |  |  |  |  |  |
| --- | --- | --- | --- | --- | --- |
| stan::gm::program\_grammar< Iterator >::program\_grammar | ( | const std::string & | *model\_name* | ) |  |

## Member Data Documentation

template<typename Iterator >

boost::spirit::qi::rule<Iterator, std::vector<var\_decl>), whitespace\_grammar<Iterator> > stan::gm::program\_grammar< Iterator >::data\_var\_decls\_r

Definition at line 45 of file program\_grammar.hpp.

template<typename Iterator >

boost::spirit::qi::rule<Iterator, std::pair<std::vector<var\_decl>, std::vector<statement> >), whitespace\_grammar<Iterator> > stan::gm::program\_grammar< Iterator >::derived\_data\_var\_decls\_r

Definition at line 51 of file program\_grammar.hpp.

template<typename Iterator >

boost::spirit::qi::rule<Iterator, std::pair<std::vector<var\_decl>, std::vector<statement> >), whitespace\_grammar<Iterator> > stan::gm::program\_grammar< Iterator >::derived\_var\_decls\_r

Definition at line 57 of file program\_grammar.hpp.

template<typename Iterator >

std::stringstream stan::gm::program\_grammar< Iterator >::error\_msgs\_

Definition at line 32 of file program\_grammar.hpp.

template<typename Iterator >

expression\_grammar<Iterator> stan::gm::program\_grammar< Iterator >::expression\_g

Definition at line 35 of file program\_grammar.hpp.

template<typename Iterator >

functions\_grammar<Iterator> stan::gm::program\_grammar< Iterator >::functions\_g

Definition at line 38 of file program\_grammar.hpp.

template<typename Iterator >

boost::spirit::qi::rule<Iterator, std::pair<std::vector<var\_decl>, std::vector<statement> >), whitespace\_grammar<Iterator> > stan::gm::program\_grammar< Iterator >::generated\_var\_decls\_r

Definition at line 63 of file program\_grammar.hpp.

template<typename Iterator >

std::string stan::gm::program\_grammar< Iterator >::model\_name\_

Definition at line 30 of file program\_grammar.hpp.

template<typename Iterator >

boost::spirit::qi::rule<Iterator, statement(), whitespace\_grammar<Iterator> > stan::gm::program\_grammar< Iterator >::model\_r

Definition at line 68 of file program\_grammar.hpp.

template<typename Iterator >

boost::spirit::qi::rule<Iterator, std::vector<var\_decl>), whitespace\_grammar<Iterator> > stan::gm::program\_grammar< Iterator >::param\_var\_decls\_r

Definition at line 73 of file program\_grammar.hpp.

template<typename Iterator >

boost::spirit::qi::rule<Iterator, program(), whitespace\_grammar<Iterator> > stan::gm::program\_grammar< Iterator >::program\_r

Definition at line 79 of file program\_grammar.hpp.

template<typename Iterator >

statement\_grammar<Iterator> stan::gm::program\_grammar< Iterator >::statement\_g

Definition at line 37 of file program\_grammar.hpp.

template<typename Iterator >

var\_decls\_grammar<Iterator> stan::gm::program\_grammar< Iterator >::var\_decls\_g

Definition at line 36 of file program\_grammar.hpp.

template<typename Iterator >

variable\_map stan::gm::program\_grammar< Iterator >::var\_map\_

Definition at line 31 of file program\_grammar.hpp.

---

The documentation for this struct was generated from the following file:

- src/stan/gm/grammars/program\_grammar.hpp


---

[ Stan Home Page ]

*© 2011–2014,
Stan Development Team.*
